# Minimally toxic tetrodotoxin analogs function as rapid chemical warning signals in pufferfish larvae

**DOI:** 10.1016/j.isci.2026.117080

**Published:** 2026-08-18

**Authors:** Yui Kaneko, Keishiro Inahashi, Inori Watanabe, Taiki Okabe, Nao Shiratori, Kyoko Shirai, Ryo Yonezawa, Rei Suo, Hajime Matsubara, Nobuo Suzuki, Masaatsu Adachi, Hideki Abe, Toshio Nishikawa, Shuichi Asakawa, Shiro Itoi

**Affiliations:** 1 College of Bioesource Sciences, Nihon University, Fujisawa, Kanagawa 252-0880, Japan; 2 Department of Aquatic Bioscience, Graduate School of Agricultural and Life Sciences, The University of Tokyo, Tokyo 113-8657, Japan; 3 Niigata Prefectural Kaiyo High School, Itoigawa, Niigata 949-1352, Japan; 4 Noto Center for Fisheries Science and Technology, Kanazawa University, Ossaka, Noto-Cho, Ishikawa 927-0552, Japan; 5 Noto Marine Laboratory, Institute of Nature and Environmental Technology, Kanazawa University, Ogi, Noto-Cho, Ishikawa 927-0553, Japan; 6 Graduate School of Pharmaceutical Sciences, Tohoku University, Aoba, Aramaki, Aoba-ku, Sendai 980-8578, Japan; 7 Graduate School of Bioagricultural Sciences, Nagoya University, Chikusa, Nagoya 464-8601, Japan

**Keywords:** chemical defense, complementary defense, pufferfish, sensory deterrence, survival strategy, takifugu, tetrodotoxin, TTX, 5,6,11-trideoxytetrodotoxin, TDT, TTX analog, warning signal

## Abstract

Early developmental stages are exceptionally vulnerable to predation, prompting many animals to rely on maternally provisioned chemical defenses. In pufferfish (*Takifugu* spp.), the potent neurotoxin tetrodotoxin (TTX) has long been assumed to be the primary deterrent. Here, we demonstrate that predator avoidance is robustly elicited by the minimally toxic TTX analog, 5,6,11-trideoxytetrodotoxin (TDT). Crucially, we show that while specific predators ingest TTX-laden larvae, they consistently reject those containing TDT upon first contact. Through sequential predation assays controlling for social learning, we reveal that larvae are actively captured but elicit immediate regurgitation by multiple predator species when TDT is present. Although both compounds are localized within the larval epidermis, TDT provides near-absolute protection against predators that bypass actual toxicity. These findings suggest that TDT functions as a high-efficiency chemical warning signal that prompts rapid unlearned aversion. Our study highlights a complementary survival strategy during vulnerable early life stages, where lethal toxicity and sensory-mediated deterrence complement each other to maximize protection.

## Highlights

• A minimally toxic TTX analog, TDT, functions as a rapid chemical warning signal in larvae
• Predators actively capture but immediately regurgitate TDT-bearing larvae upon contact
• TTX and TDT form a complementary defense system against diverse predator species
• TDT exhibits a dual role: a danger signal for predators and a social signal for pufferfish

## Graphical abstract

**Figure:**
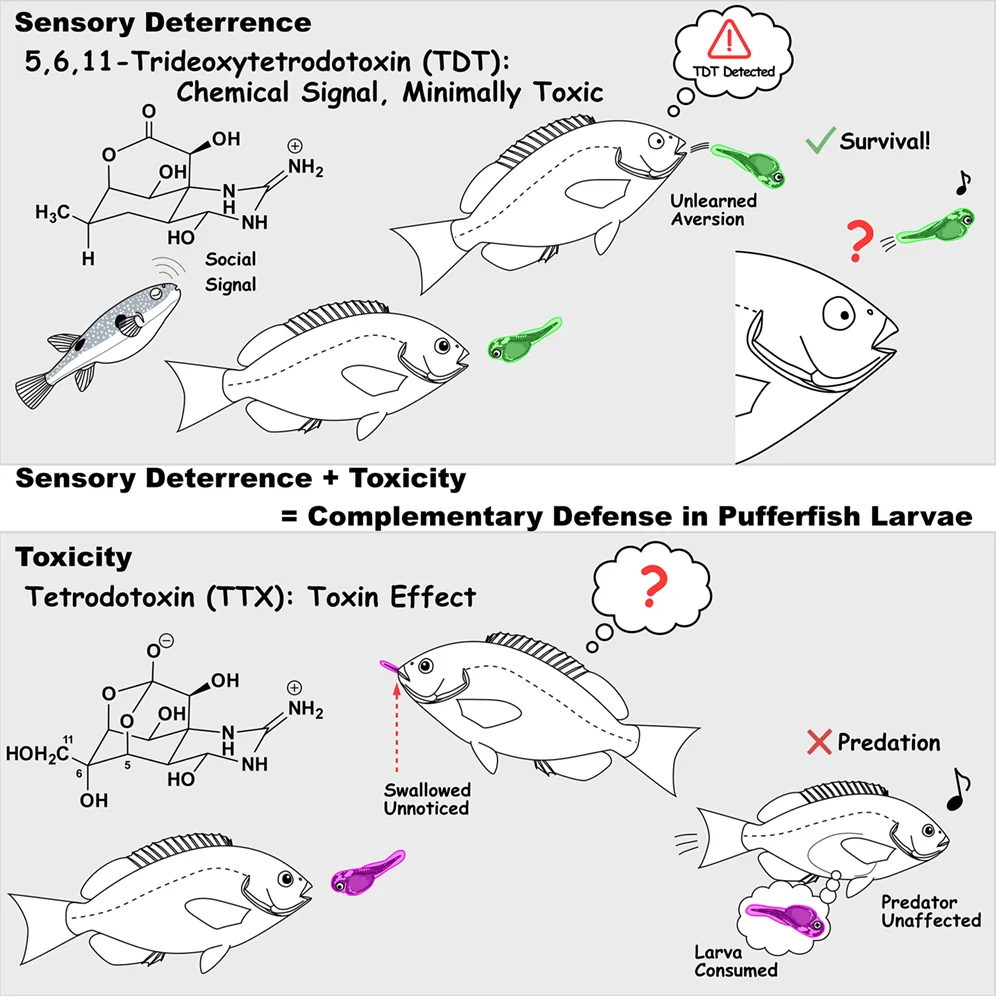

## Teaser

Zoology; Biochemistry; Biochemical toxicology; Evolutionary ecology

## Introduction

In the relentless competition for survival, organisms have evolved a staggering array of strategies to mitigate predation risk, with chemical defense representing a fundamental evolutionary innovation.^1^^,^^2^ The early stages of life history, such as eggs and newly hatched larvae, represent exceptionally vulnerable bottlenecks in the life cycle where predation pressure is most intense, due to their small size, limited mobility, and poorly developed endogenous defenses.^3^^,^^4^^,^^5^^,^^6^ To survive this critical period, diverse taxa, ranging from insects and terrestrial gastropods to amphibians, utilize maternally provisioned chemical deterrents to protect their vulnerable offspring.^7^^,^^8^^,^^9^ In marine ecosystems, the potent neurotoxin, tetrodotoxin (TTX), has long served as the prototypical example of such a lethal chemical defense.^10^^,^^11^ A major milestone in our understanding of this system was the discovery recently that pufferfish (*Takifugu* spp.) mothers actively protect their offspring through the maternal transfer of TTX to eggs and larvae.^12^ Since this pioneering discovery, the prevailing paradigm in chemical ecology has been that the survival of these vulnerable larvae depends primarily on the physiological toxicity of TTX, which acts as a lethal deterrent against potential predators.^13^^,^^14^

However, this toxicity-centric view may be incomplete, as it fails to account for the ecological roles of structural analogs that co-occur with the principal toxin. From an evolutionary perspective, relying solely on lethal toxicity presents a classic problem: the deterrent effect may occur too late to save the prey. However, if a prey species also carries a chemical warning signal that elicits immediate rejection upon oral contact, it may survive the attack before toxicity becomes necessary.^5^^,^^15^^,^^16^^,^^17^ One prominent analog, 5,6,11-trideoxytetrodotoxin (TDT), is minimally toxic due to its lack of high-affinity binding to voltage-gated sodium channels.^18^ Despite its lack of lethality, TDT was first identified in pufferfish over three decades ago^19^ and is frequently detected in various TTX-bearing organisms, such as the flatworm *Planocera multitentaculata*, which serves as a major dietary source of toxins for pufferfish.^20^^,^^21^^,^^22^^,^^23^^,^^24^^,^^25^^,^^26^ Recent studies have revealed that pufferfish are attracted by the odor of TDT but show no response to TTX itself.^27^^,^^28^ While TDT is now recognized as an olfactory social signal for adult pufferfish, its contribution to larval defense, the most vulnerable stage of the life cycle, has remained an evolutionary enigma.

In this study, we clarify the functional roles of actual toxicity and sensory deterrence in larval survival. Through controlled predation experiments using the nibbler *Girella punctata* and Japanese flounder *Paralichthys olivaceus*, we demonstrate that predator avoidance in pufferfish larvae is primarily elicited by the minimally toxic analog TDT. Combined with precise LC-MS/MS quantification and whole-mount immunohistochemistry, we reveal that TDT functions as a rapid chemical warning signal. Based on these highly uniform behavioral rejections, we hypothesize that TDT may act as a “sensory proxy” that exploits pre-existing predator sensitivities to the TTX scaffold.^29^ Rather than an evolutionary shift that entirely replaces actual toxicity, our findings propose a complementary defensive strategy: TTX provides baseline lethal defense against certain predators, while TDT complements it by triggering immediate sensory aversion in others. This dual system provides a new perspective on how organisms utilize structural analogs to optimize defensive investments during their most vulnerable life stages.

## Results

### Effects of TTX and TDT on predator behavior

To evaluate the defensive efficacy of TTX and TDT, we conducted two sets of predation experiments. Crucially, across all trials, video recordings confirmed that surviving larvae were not simply ignored by the predators. Rather, predators actively captured the larvae but immediately regurgitated them upon oral contact (Videos S1, S2, S3, S4, S5, S6, S7, S8, S9, S10, S11, S12, S13, S14, S15, and S16). Thus, “survival” in our assays strictly represents successful chemical deterrence leading to rapid oral rejection.

Video S1. Predation on larvae from a TTX-treated parent by nibbler Girella punctata (Trial 1) The video shows two individuals of G. punctata preying on larvae derived from a parent pufferfish treated with TTX

Video S2. Rejection of larvae from a TDT-treated parent by nibbler Girella punctata (Trial 1) The video shows two individuals of G. punctata attempting to prey on, and subsequently spitting out, larvae derived from a parent pufferfish treated with TDT

Video S3. Predation on larvae from an acetic acid-treated parent by nibbler Girella punctata (Trial 1) The video shows two individuals of G. punctata preying on larvae derived from a parent pufferfish treated with acetic acid

Video S4. Predation on larvae from an untreated parent by nibbler Girella punctata (Trial 1) The video shows two individuals of G. punctata preying on larvae derived from an untreated parent pufferfish

Video S5. Rejection of larvae from a TTX-treated parent by Japanese flounder *Paralichthys olivaceus* (Trial 2) The video shows five individuals of *P. olivaceus* attempting to prey on, and subsequently spitting out, larvae derived from a parent pufferfish treated with TTX

Video S6. Rejection of larvae from a TDT-treated parent by Japanese flounder *Paralichthys olivaceus* (Trial 2) The video shows five individuals of *P. olivaceus* attempting to prey on, and subsequently spitting out, larvae derived from a parent pufferfish treated with TDT

Video S7. Predation on larvae from an acetic acid-treated parent by Japanese flounder *Paralichthys olivaceus* (Trial 2) The video shows five individuals of *P. olivaceus* preying on larvae derived from a parent pufferfish treated with acetic acid

Video S8. Predation on larvae from an untreated parent by Japanese flounder *Paralichthys olivaceus* (Trial 2) The video shows five individuals of *P. olivaceus* preying on larvae derived from an untreated parent pufferfish

Video S9. Predation on larvae from a TTX-treated parent by a nibbler Girella punctata (Trial 3) The video shows a single individual of G. punctata preying on larvae derived from a parent pufferfish treated with TTX

Video S10. Rejection of larvae from a TDT-treated parent by a nibbler Girella punctata (Trial 3) The video shows a single individual of G. punctata attempting to prey on, and subsequently spitting out, larvae derived from a parent pufferfish treated with TDT

Video S11. Rejection of larvae from a TTX and TDT-treated parent by a nibbler Girella punctata (Trial 3) The video shows a single individual of G. punctata attempting to prey on, and subsequently spitting out, larvae derived from a parent pufferfish treated with both TTX and TDT

Video S12. Predation on larvae from an untreated parent by a nibbler Girella punctata (Trial 3) The video shows a single individual of G. punctata preying on larvae derived from an untreated parent pufferfish

Video S13. Rejection of larvae from a TTX-treated parent by a Japanese flounder *Paralichthys olivaceus* (Trial 4) The video shows a single individual of *P. olivaceus* attempting to prey on, and subsequently spitting out, larvae derived from a parent pufferfish treated with TTX

Video S14. Rejection of larvae from a TDT-treated parent by a Japanese flounder *Paralichthys olivaceus* (Trial 4) The video shows a single individual of *P. olivaceus* attempting to prey on, and subsequently spitting out, larvae derived from a parent pufferfish treated with TDT

Video S15. Rejection of larvae from a TTX and TDT-treated parent by a Japanese flounder *Paralichthys olivaceus* (Trial 4) The video shows a single individual of *P. olivaceus* attempting to prey on, and subsequently spitting out, larvae derived from a parent pufferfish treated with both TTX and TDT

Video S16. Predation on larvae from an untreated parent by a Japanese flounder *Paralichthys olivaceus* (Trial 4) The video shows a single individual of *P. olivaceus* preying on larvae derived from an untreated parent pufferfish

**Figure 1:**
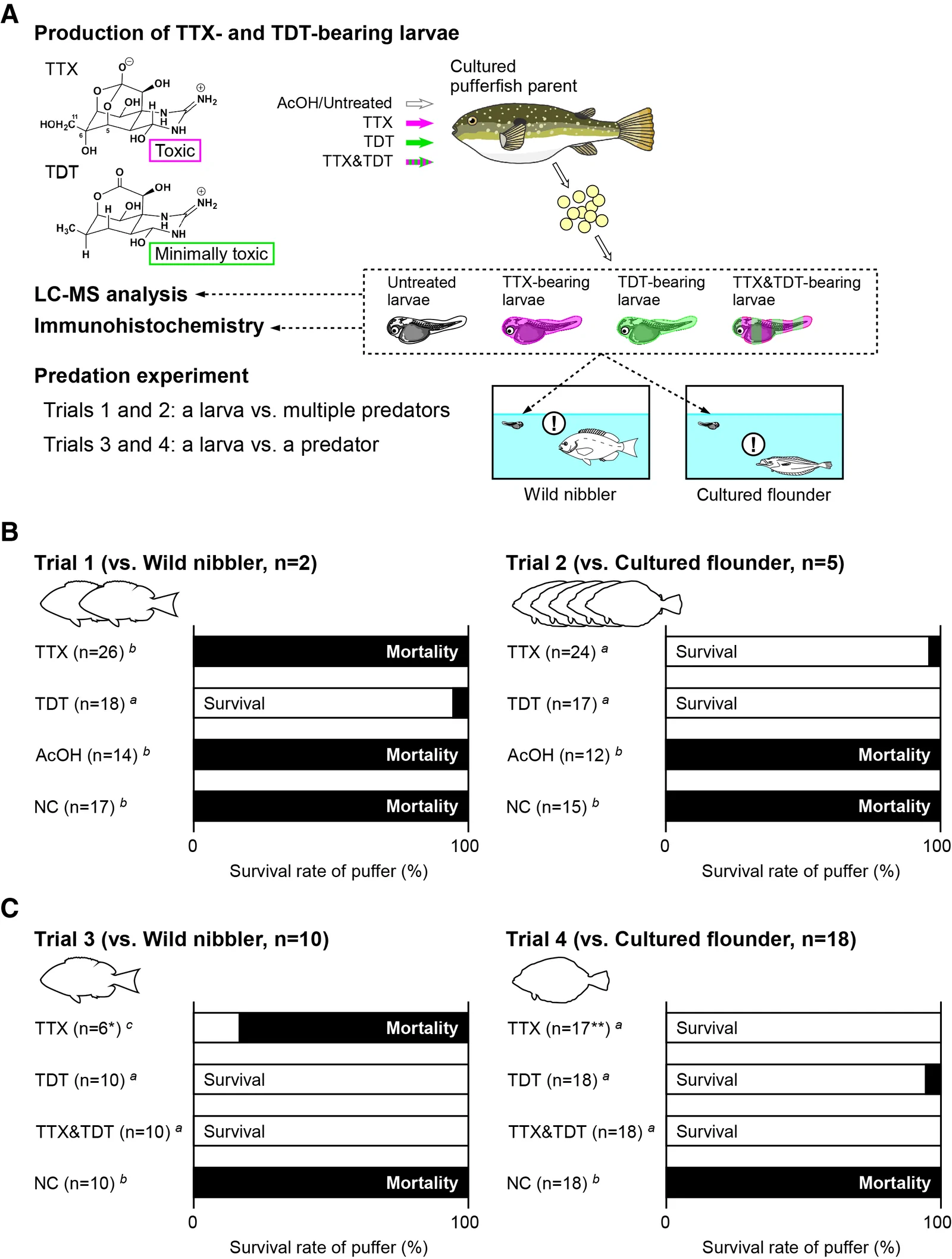
Protective effects of TTX and 5,6,11-trideoxyTTX (TDT) in pufferfish larvae against predators (A) Production of pufferfish larvae containing TTX, TDT, or both compounds. The larvae were subjected to LC-MS analysis, immunohistochemistry, and predation assays. In Trials 1 and 2, multiple predators (two *G. punctata* or five *P. olivaceus*) were housed in a single container to rapidly induce predatory behavior and evaluate overall survival, while in Trials 3 and 4, one predator was used per larva. (B) Effects of parental TTX and TDT administration on larval survival when exposed to predators (Trials 1 and 2). The left panel shows results using wild nibbler juveniles as predators, and the right panel shows results using cultured flounder juveniles. TTX, TDT, AcOH, and NC indicate larvae derived from TTX-administered, TDT-administered, acetic acid (AcOH)-administered, and untreated parents, respectively. Numbers in parentheses indicate the number of larvae tested. (C) Effects of parental TTX and TDT administration on larval survival when exposed to predators (Trials 3 and 4). The left panel shows results using wild nibbler juveniles as predators, and the right panel shows results using cultured flounder juveniles. Numbers in parentheses next to compound names indicate the number of larvae tested (equal to the number of predators). TTX, TDT, TTX&TDT, and NC indicate larvae derived from TTX-administered, TDT-administered, both compound-administered, and untreated parents, respectively. In 4 out of 10 trials (∗) and 1 out of 18 trials (∗∗), the larvae were simply ignored by the predators, and thus no predatory behavior was observed. A significant difference in survival rate was detected by pairwise Fisher’s exact test with Holm correction (adjusted *p* < 0.001 for ^*a*^ vs. ^*b*^, adjusted *p* < 0.01 for ^*a*^ vs. ^*c*^).

We first conducted an initial screening (Trials 1 and 2) using multiple predators per container to rapidly induce feeding behavior against four types of *T. alboplumbeus* larvae: untreated, acetic acid (AcOH)-administered (used as a vehicle procedural control), TTX-administered, and TDT-administered (Figures 1A and 1B). While this setup effectively stimulated predation, it could potentially introduce confounding factors such as social learning (e.g., fish observing tank mates regurgitating). To eliminate this social context and rigorously assess individual responses, we subsequently conducted Trials 3 and 4 using a single predator per container against untreated, TTX-bearing, TDT-bearing, and TTX+TDT co-bearing larvae (Figures 1A and 1C). Both sets of experiments yielded highly consistent results.

In the assays using the nibbler *G. punctata* (Trials 1 and 3), TDT-bearing and TTX+TDT-bearing larvae exhibited nearly complete survival (adjusted *p* < 0.001 vs. controls; Table S1). In contrast, TTX alone provided minimal defense, with survival rates comparable to negative controls. The nibblers consistently rejected larvae containing TDT upon first contact but readily consumed those containing only TTX. Conversely, in the assays using Japanese flounder *P. olivaceus* (Trials 2 and 4), both TDT and TTX were highly effective, resulting in near-absolute survival in all compound-containing groups (adjusted *p* < 0.001). These results demonstrate that while TTX is effective only against specific predators such as the flounder, TDT functions as a consistently potent deterrent across the tested species.

### Quality, quantity, and localization of TTX and TDT

**Figure 2:**
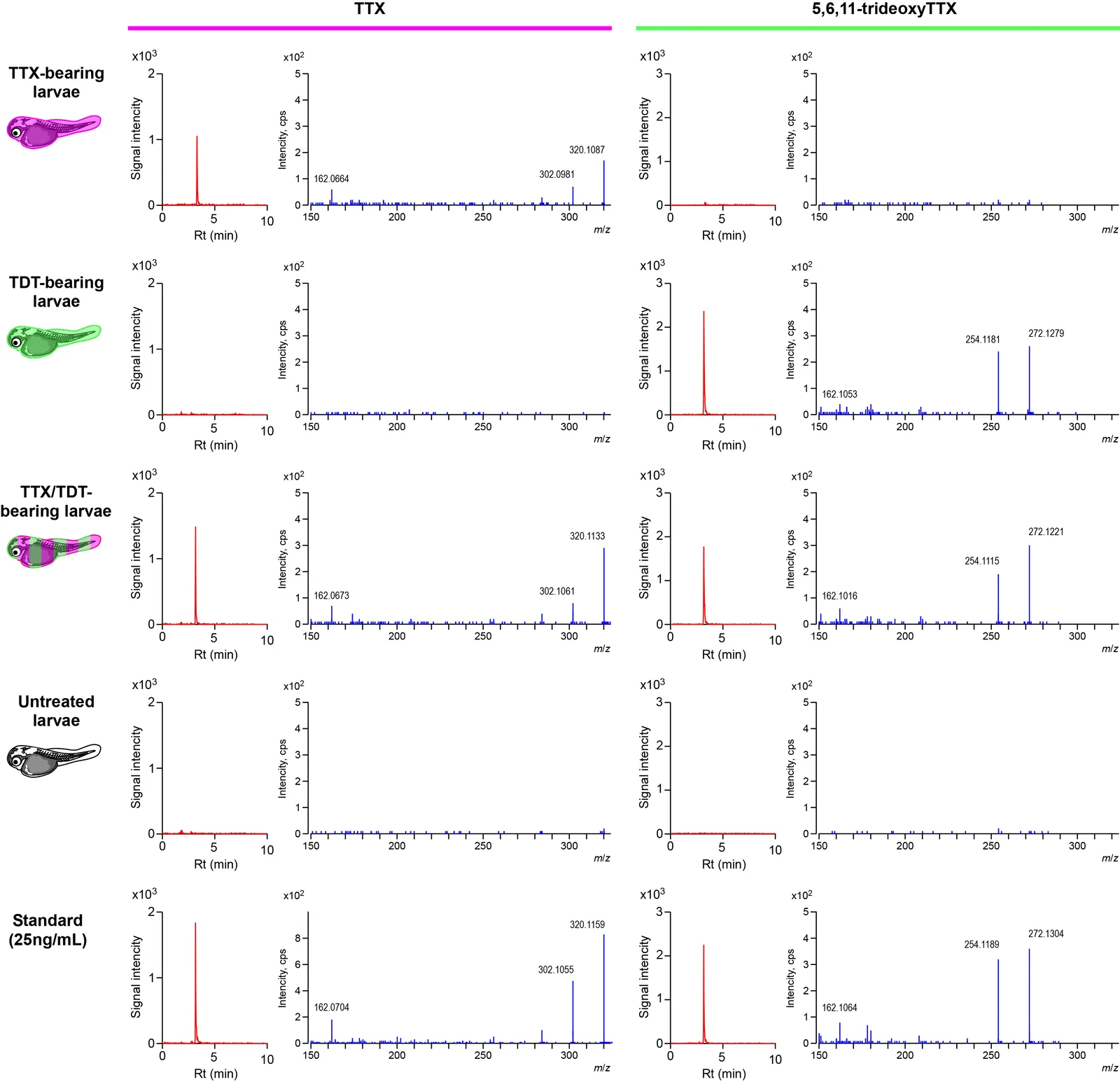
Chromatograms and mass spectra of TTX and 5,6,11-trideoxyTTX (TDT) in larvae of the pufferfish *Takifugu alboplumbeus* derived from untreated (including acetic acid-administered), TTX-administered, TDT-administered, and both compound-administered parents The left panels show the chromatographic patterns of TTX, whereas the right panels show those of TDT. The lower panels display the chromatograms of standard compounds of TTX and TDT.

LC-MS/MS analysis confirmed the chemical specificity of our experimental model: larvae from TTX-administered parents contained 6.5–10 ng/individual of TTX with no detectable TDT, whereas those from TDT-administered parents contained 11–22 ng/individual of TDT without detectable TTX (Figures 2, S1 and S2, and Table S2).

**Figure 3:**
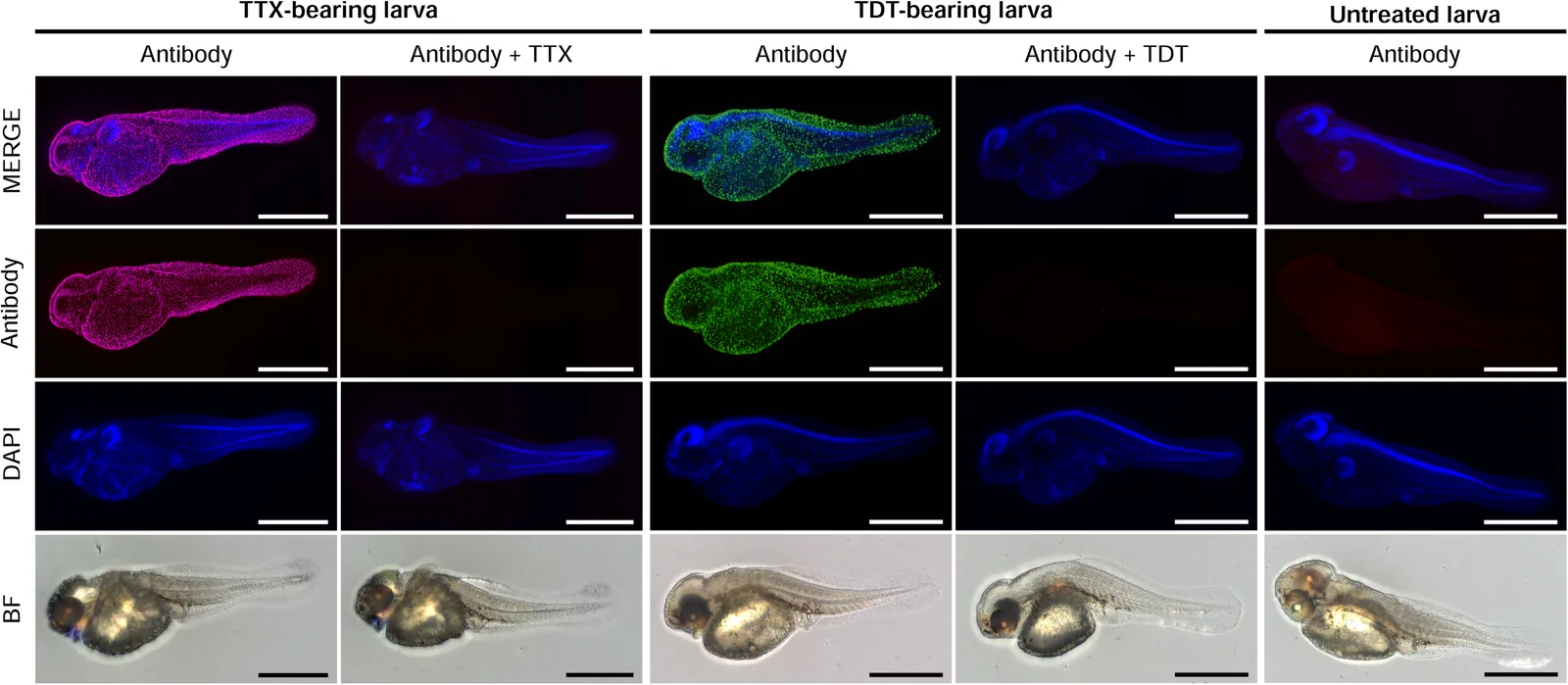
Localization of TTX and 5,6,11-trideoxyTTX (TDT) throughout the epidermis of *Takifugu alboplumbeus* larvae derived from TTX- or TDT-administered parents TTX-bearing larvae show a magenta signal (indicating TTX localization) and TDT-bearing larvae show a green signal (indicating TDT localization). Both signals were observed throughout the epidermis. TTX was detected using an anti-TTX monoclonal antibody. TDT was detected using the same anti-TTX monoclonal antibody, which exhibits weak, albeit detectable, cross-reactivity with TDT. In control experiments, the specific signal was abolished by pre-absorption of the antibody with an excess amount (10,000-fold) of the respective compound (TTX or TDT). No specific signal was observed in larvae derived from untreated parents. TTX signal: Magenta signal detected by anti-TTX antibody. TDT signal: green signal detected by anti-TTX antibody cross-reacting with TDT. DAPI: DAPI staining (blue, indicating nuclei/DNA). BF: Bright-field observation. MERGE: merged image of the specific signal (TTX: magenta, TDT: green) and DAPI (blue).

To visualize maternal transfer and tissue distribution, we performed whole-mount immunohistochemistry using an anti-TTX monoclonal antibody with known weak cross-reactivity to TDT.^30^^,^^31^ Signal specificity was verified by pre-absorption competitive assays; immunoreactivity in TDT- and TTX-bearing larvae was completely abolished by pre-incubation with a 10,000-fold molar excess of the corresponding compound (Figure 3). Both compounds were localized broadly throughout the larval epidermis, consistent with a role in predator sensory detection. Independent validation by LC-MS/MS confirms that the observed epidermal distribution reflects physiological localization.

## Discussion

TTX has long been regarded as the primary lethal defense in pufferfish.^10^^,^^11^ However, our study reveals a fundamentally different defensive strategy operating during the highly vulnerable larval stage. We demonstrate that predator avoidance is primarily driven by a maternally transferred, minimally toxic analog, TDT, rather than by lethal toxicity alone. Unlike previously described chemical deterrents that are actively deployed by adult organisms,^5^^,^^15^^,^^32^^,^^33^^,^^34^^,^^35^ TDT is provisioned in the embryonic stage and confers protection through rapid, sensory-mediated oral rejection.

Chemical defenses commonly rely on learned aversion.^36^^,^^37^ In our study, cultured (naive) Japanese flounder consistently rejected TTX- and TDT-bearing larvae upon first contact, strongly supporting the existence of an unlearned, innate sensory aversion. While the nibblers used in our study were wild-caught, meaning we cannot completely rule out prior environmental exposure to TTX-related compounds, their highly uniform and immediate regurgitation of TDT suggests that this avoidance behavior is deeply rooted in predator sensory systems. The species-specific responses we observed (nibblers selectively avoided TDT, whereas flounders avoided both TTX and TDT) likely reflect differences in the tuning breadth of gustatory receptors across fish species.^38^^,^^39^^,^^40^^,^^41^^,^^42^^,^^43^

The ecological ubiquity of TDT across multiple trophic levels reinforces its potential role as a reliable chemical warning signal. Recent studies reveal that many TTX-bearing marine organisms, including toxic flatworms that serve as major dietary sources for pufferfish, sequester TDT in significantly high concentrations.^21^^,^^25^^,^^44^^,^^45^^,^^46^^,^^47^ Electrophysiological studies have shown that the gustatory system of teleost fishes can respond to TTX (although the presence of unquantified TDT in those historical samples cannot be ruled out), likely perceiving it as a bitter stimulus that shares detection pathways with known bitter substances such as quinine and strychnine.^29^^,^^48^^,^^49^ Because bitter taste is typically mediated by T2R-type receptors,^43^ it is plausible that predator sensory systems have evolved to recognize the structural scaffold of TTX/TDT. Based on these lines of evidence, we hypothesize that TDT acts as a “sensory proxy” that exploits these pre-existing predator sensitivities. By presenting this abundant and prevalent compound, pufferfish may effectively “hack” the predator’s innate threat-detection circuitry, triggering rejection without requiring the physiological lag of intoxication.

Importantly, rather than representing an evolutionary shift that replaces actual toxicity to save metabolic costs, our findings suggest a highly effective complementary defensive strategy. In natural ecosystems, pufferfish mothers provision their eggs with both TTX and TDT.^46^^,^^47^ Our data perfectly illustrate the adaptive value of this dual provisioning: TTX provides a baseline lethal defense that is effective against certain predators (e.g., flounder), but it fails to deter others (e.g., nibbler). TDT complements this vulnerability by functioning as a potent sensory deterrent, protecting larvae from predators that might otherwise tolerate or ignore actual toxicity. This dual system, combining lethal effects (TTX) with rapid sensory warning (TDT), maximizes larval survival across multiple potential predators. While our data convincingly demonstrate that TTX and TDT are major contributors to larval defense, it is highly likely that other currently unidentified chemical, physical, or behavioral factors could act synergistically with this dual system under natural ecological conditions. Indeed, the use of multiple signal modalities is a widespread strategy in predator-prey interactions. For example, many toxic insects reinforce their visual warning colors with olfactory alerting signals, such as pyrazines, engaging multiple sensory systems of predators to maximize deterrence.^32^ Such multimodal integration may similarly enhance the survival of pufferfish larvae in the wild.

Recent studies have demonstrated that pufferfish themselves utilize olfaction to detect TDT rather than TTX, implicating this compound in behaviors related to feeding and reproduction.^27^^,^^28^^,^^50^^,^^51^ Coupled with our current findings, this highlights a fascinating ecological duality of TDT. The same chemical scaffold elicits diametrically opposite behavioral valences depending on the sensory modality and the species involved: it acts as a “danger signal” to be tasted and rapidly rejected by predators, and simultaneously functions as a “social signal” to be smelled and followed by pufferfish. These findings illustrate how a single minimally toxic analog can function simultaneously as a defensive warning against predators and a social cue for conspecifics.

In conclusion, our study demonstrates that effective chemical defense does not rely solely on lethality, but functions synergistically with minimally toxic sensory cues that trigger rapid avoidance. By revealing TDT as a functional chemical warning signal, these findings broaden our understanding of predator-prey interactions. Identifying the specific molecular receptors responsible for TDT detection in predators will be a critical next step toward fully elucidating this complementary defense mechanism.

### Limitations of the study

While our behavioral and analytical data convincingly demonstrate that TDT functions as a potent sensory deterrent, the underlying receptor-level and neural mechanisms remain hypothetical at this stage. Identifying the specific molecular receptors (e.g., T2R-type bitter taste receptors) responsible for TDT detection in predators is a necessary next step. Furthermore, while the dual TTX/TDT system is a major contributor to larval defense, it is highly likely that other currently unidentified chemical, physical, or behavioral factors could act synergistically with this system under natural ecological conditions. Future field-based studies will be required to fully elucidate the multimodal integration of these defensive strategies in the wild.

## Resource availability

### Lead contact

Further information and requests for resources should be directed to and will be fulfilled by the lead contact, Shiro Itoi (sitoi@nihon-u.ac.jp).

### Materials availability

This study did not generate new, unique reagents.

### Data and code availability

• This study produced no data.
• This study produced no code.
• Any additional information required to reanalyze the data reported in this paper is available from the lead contact upon request.

## Acknowledgments

This study was supported in part by the Grant-in-Aid for Scientific Research (A) from the 10.13039/501100001691Japan Society for the Promotion of Science (JSPS) (grant nos. 19H00954, 23H00347) and the Research Grant of 10.13039/100007683Nihon University (2023–2024). We thank Dr. Kentaro Kawatsu, Osaka Prefectural Institute of Public Health, for providing the anti-TTX monoclonal antibody. We thank the students of Niigata Prefectural Kaiyo High School for their cooperation in raising the pufferfish. This work was performed under the cooperative research program of the 10.13039/100017993Institute of Nature and Environmental Technology, Kanazawa University (Accept Nos. 23032, 24025, 25027).

## Author contributions

S.I. conceived and designed the study; Y.K., I.W., T.O., N.Sh., K.S., H.M., N.Su., and S.I. collected samples; Y.K., I.W., T.O., N.Sh., K.S., R.S., H.A., and S.I. performed rearing experiments; Y.K., I.W., N.Sh., K.S., R.S., M.A., H.A., T.N., and S.I. conducted TTX/TDT extraction, purification, and LC-MS/MS analysis; K.I., R.Y., and S.A. performed immunohistochemistry; Y.K. drafted the manuscript; S.I. critically revised the manuscript. S.I. acquired funding. All authors read and approved the final manuscript.

## Declaration of interests

The authors declare no competing interests.

## STAR★Methods

### Key resources table

**Table:** 

REAGENT or RESOURCE	SOURCE	IDENTIFIER
**Antibodies**		

Mouse monoclonal anti-Tetrodotoxin (clone TX-7F)	RIKEN Cell Bank (originally developed by Kawatsu et al.^30^)	Cat#RCB1856

**Chemicals, peptides, and recombinant proteins**		

human gonadotropin	Sigma-Aldrich	Cat#CG5-1VL; CAS: 9002-61-3
Tetrodotoxin	Nacalai Tesque	Cat#32775-51; CAS: 4368-28-9
Tetrodotoxin	FUJIFILM Wako Pure Chemical	Cat#206–11071; CAS: 4368-28-9
5,6,11-Trideoxytetrodotoxin, chemically synthesized	Adachi et al.^52^	N/A
5,6,11-Trideoxytetrodotoxin, purified	This paper	N/A
Acetic acid	Kanto Chemical	Cat#01021-70; CAS: 64-19-7
Acetonitrile, HPLC grade	Sigma-Aldrich	Cat#34851; CAS: 75-05-8
Ammonium formate, HPLC grade	Kanto Chemical	Cat#01298-23; CAS: 540-69-2
Formic acid, HPLC grade	FUJIFILM Wako Pure Chemical	Cat#067–04531; CAS: 64-18-6
10×PBS(−)	FUJIFILM Wako Pure Chemical	Cat#163-25265
4% Paraformaldehyde Phosphate Buffer Solution	FUJIFILM Wako Pure Chemical	Cat#163–20145; CAS: 30525-89-4
DAPI solution	DOJINDO Laboratories	Cat#D523
Ethanol	FUJIFILM Wako Pure Chemical	Cat#057–00451; CAS: 64-17-5
VECTASHIELD Mounting Medium	Vector Laboratories	Cat#H-1000

**Critical commercial assays**		

CUBIC Trial Kit	FUJIFILM Wako Pure Chemical	Cat#290-80801
VectaFluor Excel Amplified Fluorescent Staining Kit	Vector Laboratories	Cat#DK-2594

**Experimental models: Organisms/strains**		

Pufferfish *Takifugu alboplumbeus*	Cultured specimens	N/A
Nibbler *Girella punctata*	Field collection	N/A
Japanese flounder *Paralichthys olivaceus*	Cultured specimens	N/A

**Oligonucleotides**		

Primer: TS_chr14_8812k_0.4k_F: AAAATGGAGTTAACAGCAGAGGTT	Kabir et al.^53^	N/A
Primer: TS_chr14_8812k_0.5k_R: CGTCTCCTGAAGGATTTCCA	Kabir et al.^53^	N/A

**Software and algorithms**		

R version 4.5.2	R Core Team^54^	https://cran.r-project.org/

### Experimental model and study participant details

All experiments were performed in accordance with the Japanese Government Animal Protection and Management Law (No. 105) and Japanese Government Notification on Feeding and Safekeeping of Animals (No. 6). Predation experiments were conducted under permit P24-089.

#### Production of pufferfish larvae

The pufferfish *Takifugu alboplumbeus* larvae used in this study were procured by artificial fertilization after artificial induction of maturation with 300–600 IU human gonadotropin (Sigma-Aldrich) for cultured pufferfish parents (15.5–37.8 g body weight). These pufferfish parents were obtained by artificial fertilization using wild pufferfish individuals caught from the spawning ground on Enoshima Island (Kanagawa, Japan: 35°17′52″N, 139°28′47″E) and raised with non-toxic feed for 3–4 years in an aquaculture tank. Pufferfish larvae bearing TTX and/or TDT were procured by artificial fertilization using cultured pufferfish females administered TTX and/or TDT, respectively. Administration of TTX (approx. 300 μg/individual) and/or TDT (approx. 1500 μg/individual) to female pufferfish was carried out by intraperitoneal/intramuscular injection and/or oral ingestion using agar containing commercial feed and TTX and/or TDT on multiple doses during the months prior to the spawning season. Female individuals used as parents were selected by PCR specific to sex-determining region (Figure S3) using primer set of TS_chr14_8812k_0.4k_F (5′-AAAAT GGAGT TAACA GCAGA GGTT-3′) and TS_chr14_8812k_0.5k_R (5′-CGTCT CCTGA AGGAT TTCCA-3′) according to Kabir et al.^53^

Pufferfish larvae were used as the prey against fish juveniles of non-toxic species in observation of predation behavior. The amount of TTX and/or TDT in these pufferfish larvae was quantified by liquid chromatography-tandem mass spectrometry (LC-MS/MS) analysis, and the composition of TTX and analogs was confirmed by highly sensitive LC-MS analysis.^23^ These samples were stored at −30°C until extraction of TTX and analogs for instrumental analysis. The remaining larval individuals were fixed with 4% PFA solution in phosphate buffer (pH 7.4) on the day of hatching and stored at 4°C until immunohistochemical processing.

#### Preparation of predator fish

Two fish species with different environmental backgrounds were used as predators for predation experiments: wild-caught juveniles of the nibbler (*Girella punctata*, 0.35 and 0.49 g BW, in Trial 1; 0.49 ± 0.18 g BW in Trial 3), collected from a fishing port in Itoigawa, Niigata, Japan (37°06′36″N, 137°59′51″E), and cultured (naive) Japanese flounder (*Paralichthys olivaceus*, 0.19 ± 0.02 g BW in Trial 2; 0.13 ± 0.02 g BW in Trial 4), which were reared from eggs at Niigata Prefectural Kaiyo High School, ensuring no prior exposure to TTX or its analogs.

### Method details

#### TTX and TDT for administration to female pufferfish

TTX (purity >95%) for use in administration of TTX to female parents was purchased from Nacalai Tesque, while TDT for use in administration was extracted from ovaries of the pufferfish *Takifugu rubripes*, and purified in accordance with Miyazaki et al.^50^ The absence of TTX-related compounds other than the compound in inquiry in TTX or TDT solutions was confirmed by quantitative and qualitative analysis using LC-MS, as discussed in the following.

#### Observation of predation behavior

Predation behavior was examined using ∼1-day post-hatch pufferfish (*T. alboplumbeus*) larvae as prey and juvenile fish of non-toxic species as predators, housed in plastic containers. Pufferfish larvae derived from females bearing TTX and/or TDT were used as experimental prey, while those derived from untreated parents (including acetic acid-administered parents) served as controls.

To rigorously evaluate defensive efficacy, we visually monitored and video-recorded predator responses in all trials. Crucially, we defined “survival” strictly as instances where a larva was actively captured (ingested into the oral cavity) but immediately regurgitated by the predator. Any instances where predators simply ignored or failed to strike the larvae were not classified as chemical deterrence, ensuring that survival accurately reflects sensory rejection.

We adopted a two-step experimental design to rapidly induce feeding and subsequently eliminate confounding factors. We first conducted initial screenings (Trials 1 and 2) using multiple predators (two individuals for *G. punctata* and five individuals for *P. olivaceus*, respectively) housed together in a single test container. While this group setting effectively stimulated rapid predatory behavior through competitive feeding, it introduced potential confounding effects from social learning (e.g., observing tank mates regurgitating).

To eliminate this social context and confirm innate aversion, we subsequently conducted Trials 3 and 4 using a single predator (*G. punctata* and *P. olivaceus*, respectively) housed per container. In these individual assays, a single larva was introduced to a single, previously untested predator to assess independent responses. Each predator individual was used only once per test (i.e., the number of tested larvae equals the number of independent predators), ensuring robust and statistically independent replication.

#### Extraction of TTX and its analogs for qualitative and quantitative analyses

Extracts containing TTX and/or its analogs from pufferfish larvae or tissues were prepared according to Suo et al.^23^ Pooled larvae (100 individuals) or a portion of tissue was homogenized in 0.2 M acetic acid and heated for 10 min in boiling water. After centrifugation at 14,000 × g for 20 min at 4°C, the supernatant was collected. A portion of the supernatant was filtered through a 0.22 μm membrane filter and subjected to LC-MS/MS analysis for TTX and TDT quantification.

The remaining supernatant was adjusted to pH 5.5–6.0, and loaded onto an activated charcoal column equilibrated with water. After washing the column with water, the TTX and analogs were eluted with a solution containing acetic acid/ethanol/water (2:50:49, v/v), and the recovered solution was concentrated and lyophilized. The residue was redissolved in 0.1% acetic acid and filtered before LC-MS analysis for qualification of TTX and analogs.

#### Whole-mount immunohistochemistry

In accordance with our previous study,^14^ larval samples fixed in PFA were rinsed with phosphate buffered saline (PBS, pH 7.4), and immersed in a solution containing CUBIC-1/PBS (1:1, v/v), and then immersed in a CUBIC-1 solution as per the manufacturer’s protocol. Blocking was performed with 2.5% normal horse serum (Vector Laboratories). To observe TTX-specific signals in TTX-bearing larvae, immunohistochemistry was performed using mouse anti-TTX monoclonal antibody.^30^ After washing with PBS, samples were incubated with an amplifier antibody (Vector Laboratories), washed again with PBS, and then incubated with the VectaFluor reagent (Vector Laboratories). Negative control samples were prepared by using an absorbed antibody preincubated with a high concentration of TTX (10,000-fold molar ratio to the anti-TTX antibody) instead of the unabsorbed antibody. To observe TDT-specific signals in TDT-bearing larvae, immunohistochemistry was performed using the same anti-TTX monoclonal antibody, which exhibits weak, albeit detectable, cross-reactivity with TDT.^31^ Negative controls were prepared in the same way, using an antibody absorbed with a high concentration of TDT (10,000-fold molar ratio to the anti-TTX antibody) instead of the unabsorbed antibody. The absorbed antibody was used after dilution with PBS, without post-absorption purification.

Treated samples were sealed in VECTASHIELD Vibrance Antifade Mounting Medium (Vector Laboratories) on a glass slide. Observation of immunoreactivity image was done with an All-in-One Fluorescence Microscope BZ-X810 (Keyence). The three-dimensional (3D) images were acquired using the z stack and sectioning functions available with the microscope. Then, the level correction function was used to adjust the brightness levels.

### Quantification and statistical analysis

#### Quantification and qualification of TTX and analogs

The prepared extracts from the pufferfish larvae and adult tissues were approximately diluted and subjected to high-resolution LC-MS analysis according to the method of previous reports.^23^^,^^26^ TTX standard (purity 95%) was purchased from Fujifilm Wako Pure Chemical, whereas TDT standard was chemically synthesized.^52^

Quantification of TTX and TDT was carried out by LC-MS/MS analysis using a SCIEX X500R Q-TOF mass spectrometer (AB SCIEX) with an ESI source coupled to an LC-20AD solvent delivery system (Shimadzu). The chromatographic separation was performed using an Atlantis HILIC Silica column (2.1 × 150 mm, 5 μm; Waters) with 0.1% formic acid and acetonitrile at flow rate of 0.3 mL/min at 40°C. The specific precursor, dehydrated, and product ions [M + H]^+^ were measured by LC-MS/MS analysis in multiple reaction monitoring (MRM) mode for TTX (*m*/*z* 320.1, *m*/*z* 302.1, and *m*/*z* 162.06, respectively) and TDT (*m*/*z* 272.1, *m*/*z* 254.1, and *m*/*z* 162.1, respectively). The calibration curves, generated using 1–100 ng/mL of TTX and TDT, showed good linearity and precision (TTX, *r*^2^ = 0.9979; TDT, *r*^2^ = 0.9968). The limit of detection (LOD) and limit of quantification (LOQ) were determined on the basis of the instrument detection limit (IDL) method and were calculated as 0.41 ng/mL and 0.76 ng/mL, respectively, for TTX; and 1.09 ng/mL and 3.02 ng/mL, respectively, for TDT.

Qualification of TTX and its analogs in tissues of the pufferfish was carried out by LC-MS analysis using an X500R Q-TOF mass spectrometer with an ESI source coupled to an LC-20AD solvent delivery system according to the method of previous reports.^23^^,^^26^ The chromatographic separation was performed using a TSKgel Amide-80 column (2.0 × 150 mm, 5 μm; Tosoh Bioscience) with 16 mM ammonium formate in water/acetonitrile/formic acid (30:70:0.002, v/v) at flow rate of 0.2 mL/min at 28°C.^55^ The specific precursor ions [M + H]^+^ were measured by high-resolution LC-MS analysis using the corresponding extracted-ion chromatography ions (±0.02) for TDT at *m*/*z* 272.1241, dideoxyTTXs at *m*/*z* 288.1190, 11-norTTX-6(*S*)-ol at *m*/*z* 290.0983, deoxyTTXs at *m*/*z* 304.1139, and TTX and 4-*epi*TTX at *m*/*z* 320.1088. The calibration curves, generated using 1–100 ng/mL of TTX and TDT, showed good linearity and precision (TTX, *r*^2^ = 0.9977; TDT, *r*^2^ = 0.9933). The LOD, on the basis of the IDL method, was determined as 3.28 and 3.93 ng/mL for TTX and TDT, respectively.

#### Statistical analysis

Survival outcomes of *T. alboplumbeus* larvae were compared across treatment groups using Fisher’s exact tests for categorical data. For all experiments (Trials 1–4), associations between treatment groups and survival outcomes (survived vs. mortality) were evaluated using contingency tables. Fisher’s exact test was applied to the 4 × 2 tables to determine global significance across the groups: Trials 1 and 2 (Negative control, AcOH, TDT, and TTX), and Trials 3 and 4 (Negative control, TDT, TTX, and the combination of TTX and TDT). When significant differences were detected among groups, post-hoc pairwise comparisons were performed between each treatment group and the negative control. To maintain statistical rigor and account for multiple testing, *p*-values were adjusted using the Holm correction. All statistical analyses were performed using R version 4.5.2.^54^ The statistical validity is further supported by the high behavioral uniformity documented in the accompanying video data (Videos S1, S2, S3, S4, S5, S6, S7, S8, S9, S10, S11, S12, S13, S14, S15, and S16). Significance was assessed at an alpha level of 0.05.
