# Supplementary material for: Minimally toxic tetrodotoxin analogs function as rapid chemical warning signals in pufferfish larvae
Source: iScience. 2026 Aug 18;29(9):117080. doi: 10.1016/j.isci.2026.117080 (PMC13594433; doi:10.1016/j.isci.2026.117080)
Supplement: Supplementary file 1 [file mmc1.pdf]

iScience, Volume *29*

Supplemental information

Minimally toxic tetrodotoxin analogs function as rapid chemical warning signals in pufferfish larvae

Yui Kaneko, Keishiro Inahashi, Inori Watanabe, Taiki Okabe, Nao Shiratori, Kyoko Shirai, Ryo Yonezawa, Rei Suo, Hajime Matsubara, Nobuo Suzuki, Masaatsu Adachi, Hideki Abe, Toshio Nishikawa, Shuichi Asakawa, and Shiro Itoi

Supporting Information for

Minimally toxic tetrodotoxin analogs function as rapid chemical warning signals in pufferfish larvae

Yui Kaneko [1,†], Keishiro Inahashi [2,†], Inori Watanabe [1], Taiki Okabe [3], Nao Shiratori [1], Kyoko Shirai [1], Ryo Yonezawa [1,2], Rei Suo [1], Hajime Matsubara [4], Nobuo Suzuki [5], Masaatsu Adachi [6], Hideki Abe [7], Toshio Nishikawa [7], Shuichi Asakawa [2], Shiro Itoi [1,*]

[1] College of Bioesource Sciences, Nihon University, Fujisawa, Kanagawa 252-0880, Japan

[2] Department of Aquatic Bioscience, Graduate School of Agricultural and Life Sciences, The University of Tokyo, Tokyo 113-8657, Japan

[3] Niigata Prefectural Kaiyo High School, Itoigawa, Niigata 949-1352, Japan

[4] Noto Center for Fisheries Science and Technology, Kanazawa University, Ossaka, Noto-Cho, Ishikawa 927-0552, Japan

[5] Noto Marine Laboratory, Institute of Nature and Environmental Technology, Kanazawa University, Ogi, Noto-Cho, Ishikawa 927-0553, Japan

[6] Graduate School of Pharmaceutical Sciences, Tohoku University, Aoba, Aramaki, Aoba-ku, Sendai 980-8578, Japan

[7] Graduate School of Bioagricultural Sciences, Nagoya University, Chikusa, Nagoya 464-8601, Japan

†These authors equally contributed to this study.

*Correspondence: sitoi@nihon-u.ac.jp

This PDF file includes:

Figures S1 to S3

Tables S1 to S2

Legends for Movies S1 to S16

Other supporting materials for this manuscript include the following:

Movies S1 to S16

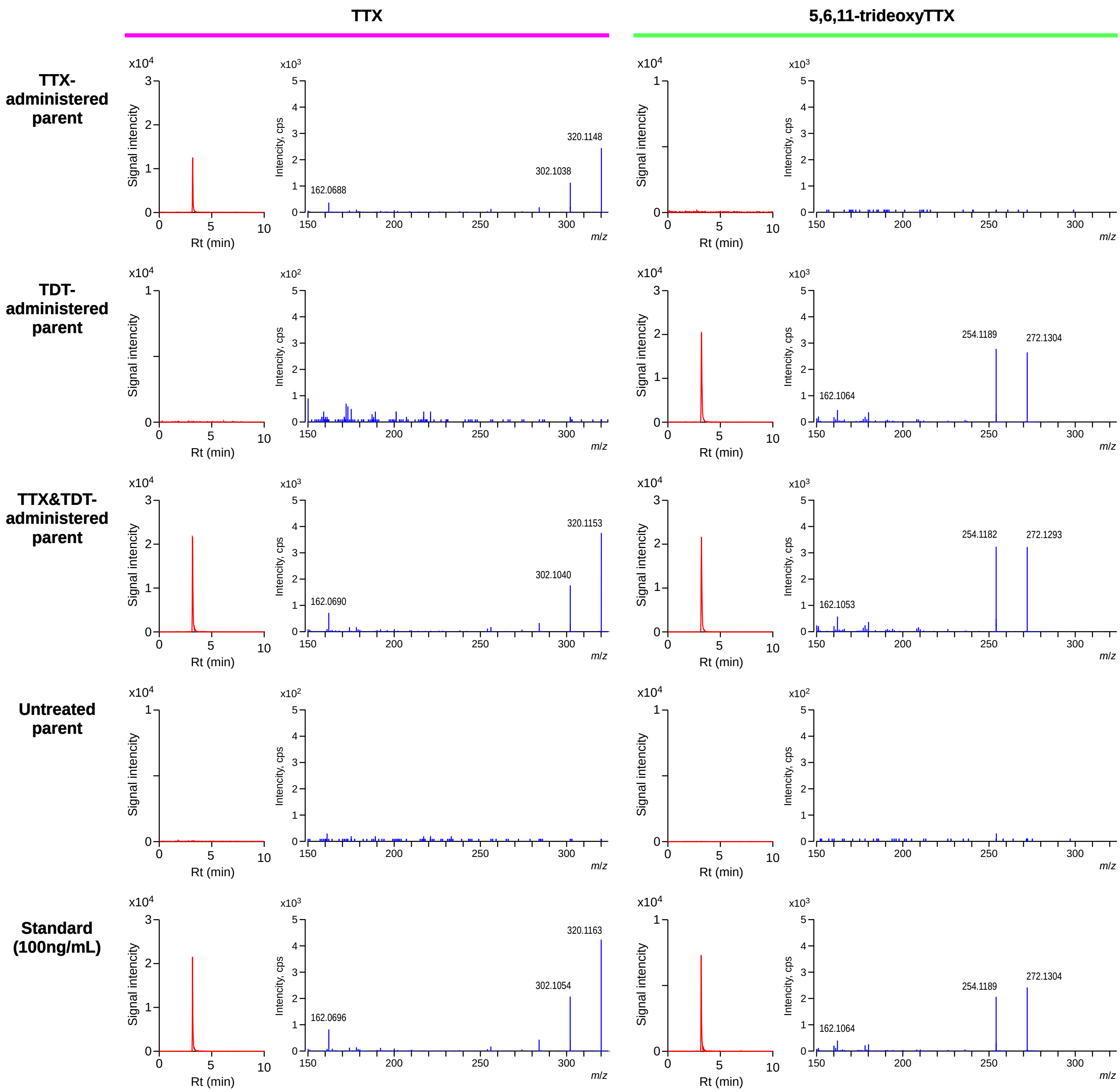

Figure S1. Chromatograms and mass spectra of TTX and 5,6,11-trideoxyTTX (TDT) in ovaries from TTX-administered, TDT-administered, both-compound-administered, and untreated (including acetic-acid-administered) parents of the pufferfish *Takifugu alboplumbeus*. The left panels show the chromatographic profiles of TTX, whereas the right panels show those of TDT. The lower panels display chromatograms of the standard compounds of TTX and TDT.

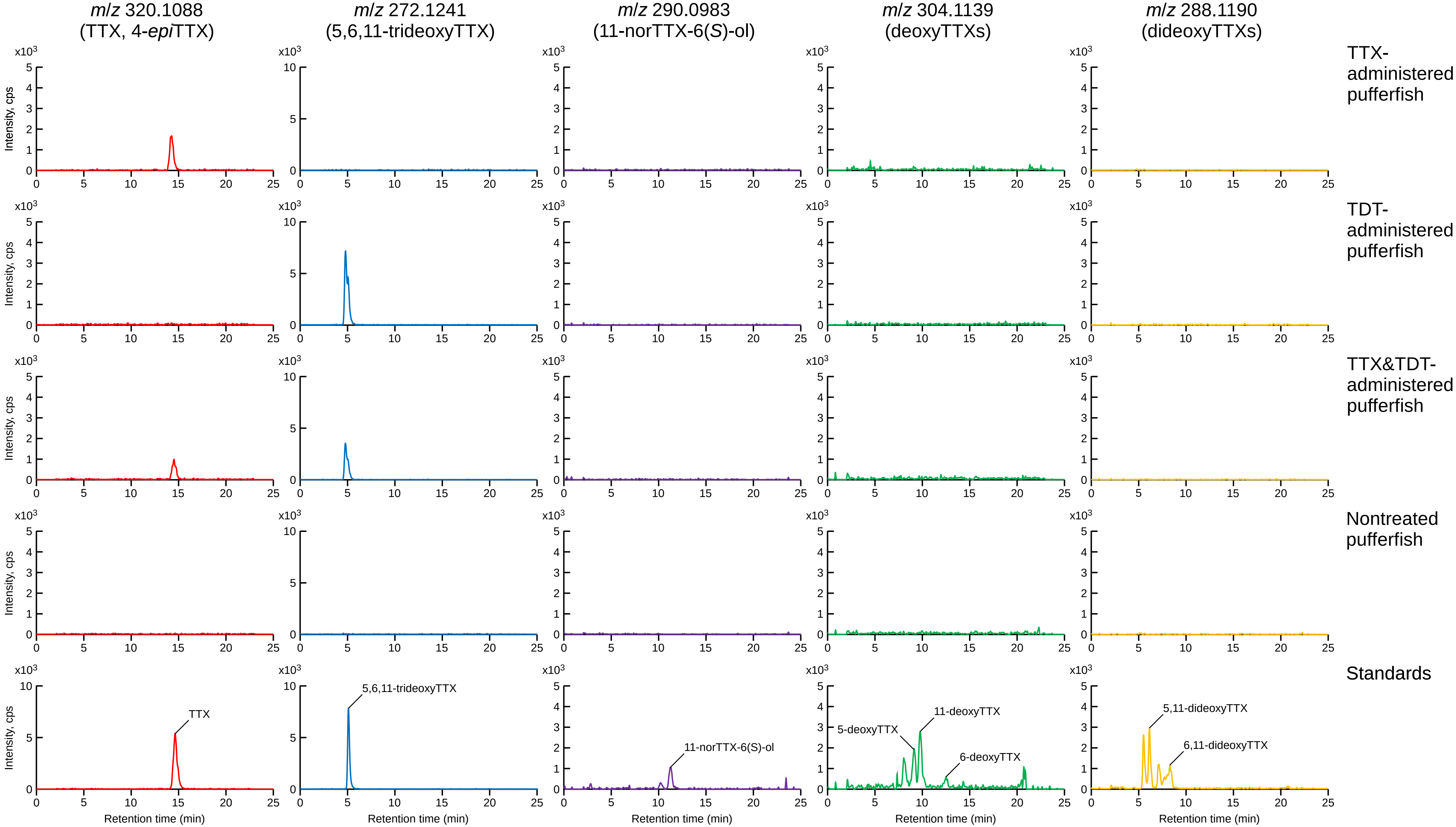

Figure S2. Comparison of extracted ion chromatograms for the analysis of TTX and its analogs in ovaries from TTX-, TDT-, and both-compound-administered parents, as well as in nontreated parents, and standards of TTX and its analogs. The panels show chromatograms for *m*/*z* 320.1088 (TTX and 4-*epi*TTX); *m*/*z* 272.1241 (TDT); *m*/z 290.0983 (11-norTTX-6(*S*)-ol); *m*/*z* 304.1139 (deoxyTTXs); and *m*/*z* 288.1190 (dideoxyTTXs). cps, counts per second.

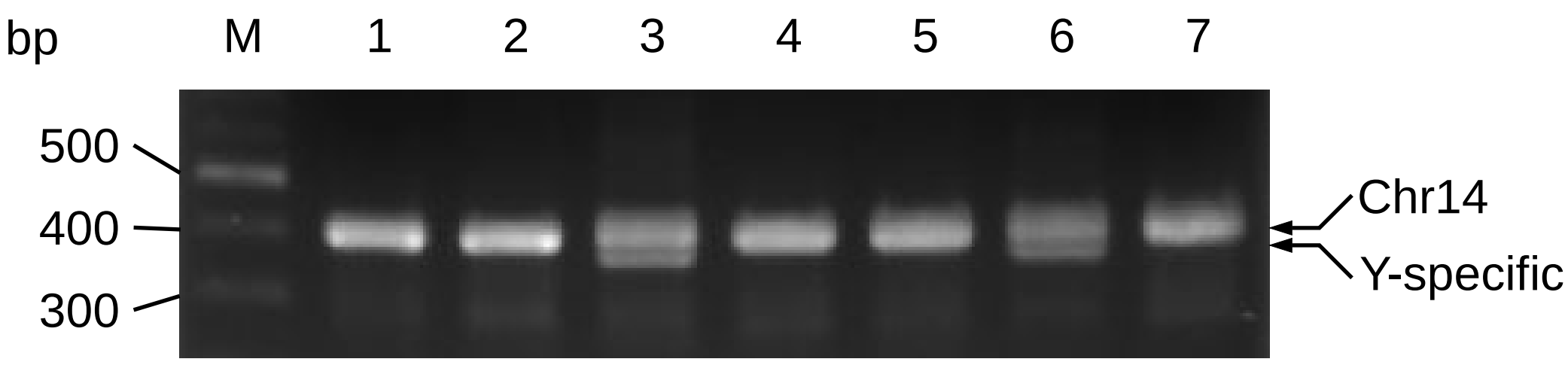

Figure S3. Typical electrophoretic patterns of PCR specific to the sex-determining region of the pufferfish *Takifugu alboplumbeus*, used for the selection of female individuals. Theoretical DNA fragment sizes for chromosome 14 (Chr14) and the Y-specific chromosome (Y-specific) are 408 bp and 352 bp, respectively. Lanes 3 and 6 were identified as male, whereas lanes 1, 2, 4, 5, and 7 were identified as female in this study.

Table S1. Pairwise comparisons of rejection and swallowing responses in predator fish trials

Trial	Comparison	Raw *P*	Adjusted *P* (Holm)[1]	Significance[2]
Trial 1	NC vs. AcOH	> 0.999	> 0.999	ns
	NC vs. TTX	> 0.999	> 0.999	ns
	NC vs. TDT	4.19×10^{-9}	2.09×10^{-8}	***
	AcOH vs. TTX	> 0.999	> 0.999	ns
	AcOH vs. TDT	3.18×10^{-8}	1.27×10^{-7}	***
	TTX vs. TDT	2.62×10^{-11}	1.57×10^{-10}	***
Trial 2	NC vs. AcOH	> 0.999	> 0.999	ns
	NC vs. TTX	3.98×10^{-11}	2.39×10^{-10}	***
	NC vs. TDT	1.77×10^{-9}	7.07×10^{-9}	***
	AcOH vs. TTX	7.99×10^{-10}	3.99×10^{-9}	***
	AcOH vs. TDT	1.93×10^{-8}	5.78×10^{-8}	***
	TTX vs. TDT	> 0.999	> 0.999	ns
Trial 3	NC vs. TTX	0.375	0.75	ns
	NC vs. TDT	1.08×10^{-5}	6.50×10^{-5}	***
	NC vs. TTX&TDT	1.08×10^{-5}	6.50×10^{-5}	***
	TTX vs. TDT	1.37×10^{-3}	5.49×10^{-3}	**
	TTX vs. TTX&TDT	1.37×10^{-3}	5.49×10^{-3}	**
	TDT vs. TTX&TDT	> 0.999	> 0.999	ns
Trial 4	NC vs. TTX	4.19×10^{-9}	1.67×10^{-8}	***
	NC vs. TDT	2.20×10^{-10}	1.32×10^{-9}	***
	NC vs. TTX&TDT	2.20×10^{-10}	1.32×10^{-9}	***
	TTX vs. TDT	> 0.999	> 0.999	ns
	TTX vs. TTX&TDT	> 0.999	> 0.999	ns
	TDT vs. TTX&TDT	> 0.999	> 0.999	ns

[1] *P*-values were calculated using Fisher's exact test and adjusted for multiple comparisons using the Holm method.

[2] Significance levels: ***$P < 0.001$, ** $P < 0.01$, ns: not significant ($P > 0.05$).

Table S2. Levels of TTX and 5,6,11-trideoxyTTX (TDT) in the cultured pufferfish *Takifugu alboplumbeus* used in this study

Trial	Parent treatment (No. of individuals)	No. of spawning individual	Parent ovaries (μg/g)[1]		Larvae (ng/individual)[2]	
			TTX	TDT	TTX	TDT
1 and 2	Nontreated (5)	2	ND	ND	ND [3]	ND [3]
	AcOH-administered (5)	1	ND	ND	ND	ND
	TTX-administered (5)	2	17±7.2	ND	10 [3]	ND [3]
	TDT-administered (5)	1	ND	121±93	ND	11
3 and 4	Nontreated (5)	1	ND	ND	ND	ND
	TTX-administered (5)	1	19±12	ND	6.5	ND
	TDT-administered (5)	1	ND	123±54	ND	22
	Both compounds-administered (4)	1	61±27	82±60	6.3	13

ND: not detected.

[1] Values represent means ± standard deviation of treated individuals (4–5 individuals).

[2] Values were calculated based on the extract from 100 larvae.

[3] Data were obtained from 100 larvae collected from two parents.

Movie S1 (separate file). Predation on larvae from a TTX-treated parent by nibbler *Girella punctata* (Trial 1). The video shows two individuals of *G. punctata* preying on larvae derived from a parent pufferfish treated with TTX.

Movie S2 (separate file). Rejection of larvae from a TDT-treated parent by nibbler *Girella punctata* (Trial 1). The video shows two individuals of *G. punctata* attempting to prey on, and subsequently spitting out, larvae derived from a parent pufferfish treated with TDT.

Movie S3 (separate file). Predation on larvae from an acetic acid-treated parent by nibbler *Girella punctata* (Trial 1). The video shows two individuals of *G. punctata* preying on larvae derived from a parent pufferfish treated with acetic acid.

Movie S4 (separate file). Predation on larvae from an untreated parent by nibbler *Girella punctata* (Trial 1). The video shows two individuals of *G. punctata* preying on larvae derived from an untreated parent pufferfish.

Movie S5 (separate file). Rejection of larvae from a TTX-treated parent by Japanese flounder *Paralichthys olivaceus* (Trial 2). The video shows five individuals of *P. olivaceus* attempting to prey on, and subsequently spitting out, larvae derived from a parent pufferfish treated with TTX.

Movie S6 (separate file). Rejection of larvae from a TDT-treated parent by Japanese flounder *Paralichthys olivaceus* (Trial 2). The video shows five individuals of *P. olivaceus* attempting to prey on, and subsequently spitting out, larvae derived from a parent pufferfish treated with TDT.

Movie S7 (separate file). Predation on larvae from an acetic acid-treated parent by Japanese flounder *Paralichthys olivaceus* (Trial 2). The video shows five individuals of *P. olivaceus* preying on larvae derived from a parent pufferfish treated with acetic acid.

Movie S8 (separate file). Predation on larvae from an untreated parent by Japanese flounder *Paralichthys olivaceus* (Trial 2). The video shows five individuals of *P. olivaceus* preying on larvae derived from an untreated parent pufferfish.

Movie S9 (separate file). Predation on larvae from a TTX-treated parent by a nibbler *Girella punctata* (Trial 3). The video shows a single individual of *G. punctata* preying on larvae derived from a parent pufferfish treated with TTX.

Movie S10 (separate file). Rejection of larvae from a TDT-treated parent by a nibbler *Girella punctata* (Trial 3). The video shows a single individual of *G. punctata* attempting to prey on, and subsequently spitting out, larvae derived from a parent pufferfish treated with TDT.

Movie S11 (separate file). Rejection of larvae from a TTX and TDT-treated parent by a nibbler *Girella punctata* (Trial 3). The video shows a single individual of *G. punctata* attempting to prey on, and subsequently spitting out, larvae derived from a parent pufferfish treated with both TTX and TDT.

Movie S12 (separate file). Predation on larvae from an untreated parent by a nibbler *Girella punctata* (Trial 3). The video shows a single individual of *G. punctata* preying on larvae derived from an untreated parent pufferfish.

Movie S13 (separate file). Rejection of larvae from a TTX-treated parent by a Japanese flounder *Paralichthys olivaceus* (Trial 4). The video shows a single individual of *P. olivaceus* attempting to prey on, and subsequently spitting out, larvae derived from a parent pufferfish treated with TTX.

Movie S14 (separate file). Rejection of larvae from a TDT-treated parent by a Japanese flounder *Paralichthys olivaceus* (Trial 4). The video shows a single individual of *P. olivaceus* attempting to prey on, and subsequently spitting out, larvae derived from a parent pufferfish treated with TDT.

Movie S15 (separate file). Rejection of larvae from a TTX and TDT-treated parent by a Japanese flounder *Paralichthys olivaceus* (Trial 4). The video shows a single individual of *P. olivaceus* attempting to prey on, and subsequently spitting out, larvae derived from a parent pufferfish treated with both TTX and TDT.

Movie S16 (separate file). Predation on larvae from an untreated parent by a Japanese flounder *Paralichthys olivaceus* (Trial 4). The video shows a single individual of *P. olivaceus* preying on larvae derived from an untreated parent pufferfish.
